# Delivery of acetamiprid to tea leaves enabled by porous silica nanoparticles: efficiency, distribution and metabolism of acetamiprid in tea plants

**DOI:** 10.1186/s12870-021-03120-4

**Published:** 2021-07-16

**Authors:** Xinyi Wang, Min Yan, Jie Zhou, Wei Song, Yu Xiao, Chuanjian Cui, Wanjun Gao, Fei Ke, Jing Zhu, Zi Gu, Ruyan Hou

**Affiliations:** 1 State Key Laboratory of Tea Plant Biology and Utilization, School of Tea and Food Science & Technology, Anhui Agricultural University, Hefei, 230036 China; 2 School of Environmental Science and Engineering, Shanghai Jiao Tong University, 800 Dongchuan Road, Shanghai, 200240 China; 3 Hefei Customs District Technical Center, Safety, Anhui Key Lab of Analysis and Detection for Food, Hefei, 230022 China; 4 School of Chemical Engineering, The University of New South Wales, Sydney, 2052 NSW Australia

**Keywords:** Porous silica nanoparticles, Acetamiprid, Biological activity, Tea plant, Distribution and metabolism

## Abstract

**Background:**

Pesticide residue and its poor utilization remains problematic in agricultural development. To address the issue, a nano-pesticide has been developed by incorporating pesticide acetamiprid in porous silica nanoparticles.

**Results:**

This nano-pesticide had an acetamiprid loading content of 354.01 mg g^−1^. Testing LC_50_ value against tea aphids of the commercial preparation was three times that of the nano-pesticide. In tea seedlings (*Camellia sinensis* L.), acetamiprid was transported upward from the stem to the young leaves. On day 30, the average retained concentrations in tea leaves treated with the commercial preparation were about 1.3 times of that in the nano-pesticide preparation. The residual concentrations of dimethyl-acetamiprid in leaves for plants treated with the commercial preparation were about 1.1 times of that in the nano-pesticide preparation. Untargeted metabolomics of by LC–MS on the young leaves of tea seedlings under nano-pesticide and commercial pesticide treatments showed significant numbers of differentially expressed metabolites (*P* < 0.05 and VIP > 1). Between the nano-pesticide treatment group and the commercial preparation treatment group there were 196 differentially expressed metabolites 2 h after treatment, 200 (7^th^ day), 207 (21^st^ day), and 201 (30^th^ day) in negative ion mode, and 294 (2^nd^ h), 356 (7^th^ day), and 286 (30^th^ day) in positive ion mode. Preliminary identification showed that the major differentially expressed metabolites were glutamic acid, salicylic acid, *p*-coumaric acid, ribonic acid, glutamine, naringenin diglucoside, sanguiin H4, PG (34:2) and epiafzelechin.

**Conclusions:**

This work demonstrated that our nano-pesticide outperformed the conventional pesticide acetamiprid in terms of insecticidal activity and pesticide residue, and the absorption, transportation and metabolism of nano-pesticide in tea plant were different, which pave a new pathway for pest control in agricultural sector.

**Graphical abstract:**

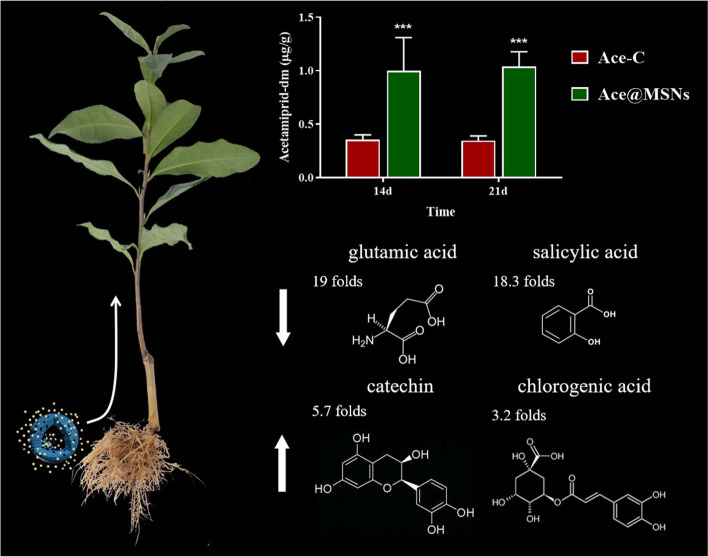

**Supplementary Information:**

The online version contains supplementary material available at 10.1186/s12870-021-03120-4.

## Background

Tea is a perennial monoculture crop, mainly growing in subtropical and tropical areas, with frequent diseases and insect pests, so year-over-year pest prevention and control are essential. Although pesticide spraying can effectively kill pests and bolster tea yield and quality, excessive spraying may lead to the presence of pesticide residues in the tea product, which will ultimately affect tea sales and harm human health. Globally, annual input of pesticides has reached 4.6 million tons. However, only about 0.1% of conventional pesticides achieve their intended pest control goals, while 99.9% diffuse into the environment and are redistributed in ecological cycles [1, 2]. With the recent and rapid development of nanotechnology, smart, nano-delivery systems have the potential to mitigate the hazards posed by traditional application of pesticides by creating slow and continuous release pesticides [3–7].

Mesoporous silica nanoparticles (MSNs) can be used effectively in many fields and are considered promising drug carriers due to their modifiable particle size, pore structure and surface functionalization [8–13]. When MSNs are synthesized, changing the concentration and properties of surfactant controls the resulting pore size, structure and particle crystallinity [14, 15]. The pore sizes of MSNs vary from 10 to 300 Å depending on the structural guide material. The use of covalent organosilane precursors and surfactants can generate a variety of silica mesophase structures, termed SBA-1 (cubic), SBA-2 (three-dimensional hexagonal), SBA-3 (hexagonal), MCM-41 (2D hexagonal), MCM-48 (cube) and MCM-50 (layered) [16].

Neonicotinoids are an important class of insecticides that function as competitive inhibitors of nicotinic acetylcholine receptors [17, 18]. Because of their unique mechanism of action, neonicotinoids have strong insecticidal activity but relatively low toxicity to mammals and non-target insects. According to their structure, neonicotinoids can be divided into three categories: neonicotinoids containing a chloropyridine heterocyclic ring (such as acetamiprid), neonicotinoids containing a chlorinated thiazole heterocyclic ring (such as thiamethoxam) and neonicotinoids containing a tetrahydrofuran heterocyclic ring (such as dinotefuran) [19]. Acetamidine, a chloropyridine heterocyclic neonicotinoid insecticide with strong absorption, wide insecticidal range and good effect, is one of the most effective insecticides against aphids, mealworms, leafhoppers, planthoppers, thrips, some small lepidopteran insects and some coleoptera pests. Acetamiprid can damage the insect gastric system and respiration system. Studies have shown that acetamiprid has higher contact toxicity to bees than acute oral toxicity (at 48 h) [20].

Metabolomics is an effective analysis method and can reveal the state of an individual organism at a given point in time. This provides valuable information about how organisms respond to internal and external disturbances, such as growth, genetic modification, disease, and environmental influences [21, 22]. In tea, the metabolites can help the tea plants resist abiotic and biotic stresses and ensure the quality of tea [23–26]. Metabolomics can identify and quantify the metabolites, metabolic pathways and regulatory mechanisms [27, 28], and has been widely used in tea research, including to screen fine tea, for tea quality evaluation [29–31], to understand how cultivation conditions induce physiological changes [32, 33], to study the metabolism of non-biological and biological stress reactions, and to outline the metabolic pathways [34–42].

At present, there are few studies on the interaction between tea plants and nano-pesticides. In this study, mesoporous silica nanoparticles (MSNs) were prepared by the sol–gel method. Acetamiprid was selected as a model pesticide to explore the feasibility of using MSNs as delivery vehicles. The insecticidal activity of acetamiprid-loaded porous silica nanoparticles (Ace@MSNs) against the tea aphid was explored. The absorption, transportation and metabolism of Ace@MSNs and a commercial preparation of acetamiprid (Ace-C) in various parts of tea plants were compared, revealing the presences of differentially expressed metabolites between the two treatments in tea leaves. Untargeted metabolomics based on LC-QTOF-MS facilitated the understanding of the absorption and transportation of the nano-pesticide in tea plants and the effects of nano-pesticides on pesticide and tea plant metabolism. The results from this study may serve as a starting point to study the interactions between pesticides, tea plants and their insect pests.

## Methods

### Chemicals and reagents

Polyvinylpolypyrrolidone (PVPP) and cetyltrimethyl ammonium bromide (CTAB) were purchased from Beijing Solarbio Science & Technology Co., Ltd. (Beijing, China). Graphitized carbon black (GCB, 120–400 Mesh), primary secondary amine (PSA, 230–400 Mesh) and C18 (230–400 Mesh, 60 Å; SiliCycle, Canada) were obtained from Shanghai ANPEL Scientific Instrument Co., Ltd. (Shanghai, China). Tetraethyl orthosilicate (TEOS), ammonium hydroxide and ethyl acetate were purchased from Yixing Wuxi Zhanwang Chemical Reagent Co., Ltd. (Yixing, China). Silane coupling agent (KH570) was purchased from Qiyi Biotechnology (Shanghai) Co., Ltd. (Shanghai, China). MgSO_4_ was purchased from Sinopharm Chemical Reagents Co., Ltd. (Shanghai, China). The commercially available silica nanoparticles (MSNs-C, > 99.5%, 50 ± 5 nm) was purchased from Shanghai McLean Biochemical Co., Ltd. (Shanghai, China). Acetamiprid (98.1%) was purchased from Dr. Ehrenstorfer (Augsburg, Germany). Acetamiprid commercial preparation (Ace-C, 70%) was purchased from Shandong Shengpeng Technology Co., Ltd. (Shandong, China).

Annual tea saplings of the *Camellia sinensis* L. cultivar Shu Cha Zao were purchased from Anhui Dechang nursery stock Co., Ltd. (Anhui, China). According to the previous research, annual tea saplings of the *Camellia sinensis* L. cultivar Shu Cha Zao was hydroponically cultured in nutrient solution until the growth rate of all tea saplings was consistent. All cultivation experiments were carried out in the greenhouse of Anhui Agricultural University (Hefei, China). The nutrient solution contained (mg L^−1^): 40 K^+^, 30 NH^4+^, 25 Mg^2+^, 20 Ca^2+^, 10 NO^3−^, 10 Al^3+^, 3.1 PO^4−^, 1.0 Mn^2+^, 0.35 Fe^2+^, 0.1 B3^+^, 0.1 Zn^2+^, 0.05 Mo^+^, 0.025 Cu^2+^.

### Synthesis of porous silica nanoparticles (MSNs)

Briefly, 100 mg of CTAB was dissolved in 100 mL distilled water in a three-mouth round-bottom flask under agitation by a magnetic rotor. The water bath was heated to 55 °C, and then 3 mL ammonium hydroxide (catalyst), 1 mL ethyl orthosilicate (silicon source) and 5 mL ethyl acetate were added dropwise. After a 6-h reaction, the mixture was cooled to room temperature and washed three times with anhydrous ethanol. The washed sample was dried overnight in an oven at 60 °C. After drying, the powder was calcined at 550 °C in a muffle furnace for 6 h. The final product was the MSNs.

### Synthesis of acetamiprid-loaded porous silica nanoparticles (Ace@MSNs)

#### Effect of solvent on drug loading

Five different polar solvents were tested, namely ultrapure water, acetonitrile, methanol, ethanol and dichloromethane were used to prepare a 20 µg mL^−1^ acetamiprid standard solution. The calcined MSN powder (10 mg) was dissolved in a 2-mL centrifuge tube with 1 mL of one solvent-amidine combination using a thermostatic oscillator (300 rpm) for 2 h, and then centrifuged. HPLC (Agilent 1260 liquid chromatograph) was used to test amidine concentrations in the supernatant.

#### Effect of time on drug loading

MSN powder (10 mg) was dissolved in a 2-mL centrifuge tube with 1 mL of a 20 µg mL^−1^ acetamiprid aqueous solution and mixed by an oscillator (300 rpm). The tubes were removed at different times for centrifugation, and the content of acetamiprid in the supernatant was detected by HPLC.

#### Effect of initial concentration on drug loading

MSN powder (10 mg) was dissolved in a 2-mL centrifuge tube with 1 mL of acetamiprid solutions of different concentrations (10, 20, 50, 100, 500, 1000, 2000, 3000, 4000 µg mL^−1^). The solution was vibrated in an oscillator (300 rpm) and taken out at different times for centrifugation. The concentration of acetamiprid in the supernatant was determined by HPLC.

Drug loading (DL) was calculated according to the following equation:

$$DL=\frac{({C}_{0}-{C}_{t})\times V}{1000\times m}\times 100\%$$

where DL is the drug loading (%) with respect to the loaded pesticide; C_0_ (µg mL^−1^) is the initial concentration of acetamiprid before adsorption; C_t_ (µg mL^−1^) is the acetamiprid concentration of the supernatant after adsorption; V (mL) is the volume of added acetamiprid solution; m (mg) is the mass of MSNs added.

### Characterization of nano-particles

An Hitachi HT7700 transmission electron microscope (TEM) was used to observe the internal structure of the sample at a voltage of 80 kV. The appearance of the sample was observed using an Hitachi S-4800 scanning electron microscope (SEM) at a voltage of 1 kV. A Nexus-670 Fourier transform infrared (FT-IR) spectrometer was used with a scanning wavelength of 4000 to 400 cm^−1^, a resolution of 4 cm^−1^ and 100 scans using a KBr tableting method to transmit spectrum.

### Effect of Ace@MSNs on tea aphid

Four treatments (acetamiprid technical drug, MSNs, Ace@MSNs, acetamiprid commercial preparation) were mixed with pure water to prepare solutions of five concentrations, such that the final concentration of effective components was 5, 10, 20, 40, and 80 mg L^−1^. Healthy and active tea aphids (species name) without spreading wings were reared (Reference) and used as test materials. Using a microdripper (Burkard Scientific Co., Ltd. England), 0.1 µL of each dosage was added to each insect. Each concentration of each treatment was tested 5 times, and each solution was dripped onto 10 tea aphids. A drop was added to both the front chest and back plate of each tea aphid, and then 10 treated tea aphids were placed in a plastic petri dish (diameter 12 cm). The plates were placed in an incubator at a constant temperature of 25 ºC. After 24 h, the survival rate by counting the number of dead aphids. The death criterion of tea aphid was that it was completely immobile when touching the feet and tentacles. The mortality of tea aphids was used to obtain the half lethal (LC_50_) dose of each agent, calucated as the mortality rate (%) = number of deaths / total number of test insects × 100% using the SPSS software (manufacturer and version).

### Transport, absorption and metabolism of Ace@MSNs in tea plant

The absorption, metabolism and distribution of the pesticide preparations in tea saplings were studied. The experiment was divided into two phases, the absorption stage (0–10 days) and the metabolic stage (10–30 days). In the absorption stage, the pesticides were added into the nutrient solution at a concentration of 10 µg mL^−1^. In the metabolic stage, the tea saplings were grown in nutrient solution containing 10 µg mL^−1^ acetamiprid pesticide until the 10th day (absorption stage), then transferred to fresh, untreated nutrient solution and allowed to grow for 30 days. During the whole experiment, 2 mL of nutrient solution was reserved during each random sampling of tea saplings. The roots were washed with clean water and dried with absorbent paper. The tea saplings were divided into root, stem, tender leaves (1 bud and 1–3 leaves) and mature leaves (except tender leaves). After weighing, the samples were freeze-dried at -80 ℃ and stored at -20 ℃.

### Extraction of tea samples for mass spectrometry

After grinding the freeze-dried sample with a mortar and pestle, 50 mg of ground sample was put into a small, self-sealing bag and stored at -80 ℃. Of the remaining sample, 300 mg was put into 50-ml centrifuge tube before 5 mL of acetonitrile was added. The mixture was subjected to ultrasonic extraction at 25 ℃ for 10 min. After this, 1.0 g sodium chloride and 1.0 g anhydrous MgSO_4_ were added to the centrifuge tube, the extract was mixed by vortex for 2 min, and then centrifuged at 2599 g for 5 min.

The supernatant (2 mL) was placed in a centrifuge tube containing 100 mg PVPP, 10 mg PSA, 40 mg GCB, 20 mg C18 and 60 mg anhydrous MgSO_4_. After 2 min of mixing, the mixture was centrifuged at 10,397 g for 10 min. The supernatant was filtered through a 0.22-µm membrane. The sample (200 µL) was taken to near dryness under nitrogen, dissolved in acetonitrile: water (5:95 = v/v), and stored at -80 ºC until HPLC–MS / MS analysis.

Another sample for metabolite detection was prepared by placing 30 mg of cryopreserved ground sample into a centrifuge tube and adding 1 mL of 70% (v/v) methanol in water, mixing by vortex for 2 min, and treating with ultrasound at 25 ℃ for 15 min. This was centrifuged at 10,397 g for 10 min, collecting the supernatant. This treatment was repeated on the pellet, and the supernatants were combined. The supernatant was filtered through a 0.22-µm membrane, and stored at -20 ℃ for metabolite detection. The quality control (QC) sample was a mixture of 20 µL of each sample.

### Apparatus conditions

#### Determination of Ace@MSNs by UPLC

A Phenomenex C18 column (250 × 4.6 mm i.d., 5 µm) was used with a solvent flow rate of 1.0 mL min^−1^. The column compartment temperature was set at 25 °C. The injection volume was 10 µL. The column was eluted with a mobile phase of distilled water (A) and methanol (B). The gradient-elution program comprised: 0–5 min, 10% B; 5–10 min, 20% B; 10–30 min, 30% B; 30–31 min, 40% B; and 31–40 min, 10% B.

#### Analysis of pesticide matrix and metabolite content by UPLC-MS/MS

An UPLC-electrospray ionization-tandem triple-quadrupole mass spectrometer was used to analyze the target compounds. For LC analysis, an Agilent Eclipse plus C18 column (50 mm × 2.1 mm and 1.8 µm particle size) was employed with a solvent flow rate of 0.3 mL min^−1^. The temperature of the column oven was set at 40 °C. The injection volume was 5 µL. The column was eluted with a mobile phase of water with 0.1% formic acid (A) and acetonitrile (B). The gradient elution procedure was set as follows: 0–0.5 min, 5% B; 0.5–1 min, 5% B; 1–2 min, 30% B; 2–2.5 min, 30% B; 2.5–5 min, 90% B; 5–5.5 min, 90% B; and 5.5–7 min, 5% B.

For mass spectrometry analysis, an Agilent 1290 UPLC-MS/MS (QQQ, Agilent Technologies, Palo Alto, CA, USA) was used in multiple reaction monitoring (MRM) mode, with the parameters: dry gas flow rate, 11 L min^−1^; dry gas temperature, 300 °C; nebulizer pressure, 15 psi. Nitrogen was used as the nebulizer and collision gas.

#### Analysis of metabolites in tea leaves by UPLC-QTOF-MS

A UPLC-Q-TOF/MS was used to analyze the target compounds. For LC analysis, an ACQUITY UPLC® HSS T3 (100 mm × 2.1 mm and 1.8 µm particle size) was employed with a solvent flow rate of 0.14 mL min^−1^. The temperature of the column oven was set at 40 °C. The injection volume was 1 µL. The column was eluted with a mobile phase of water with 0.1% formic acid (A) and methanol (B). The gradient elution procedure was set as follows: 0–0.1 min, 5% B; 0.1–13 min, 5% B; 13–16 min, 95% B; 16–16.1 min, 95% B; 16.1–21 min, 5% B. For the mass spectrometry analysis, an Agilent time-of-flight mass spectrometer (Agilent Technologies, Palo Alto, CA, USA) was used in multiple reaction monitoring (MRM) mode, with the parameters: dry gas flow rate, 8 L min^−1^; dry gas temperature, 320 °C; nebulizer pressure, 35 psi; sheath temperature, 350 °C; sheath gas flow, 11 L min^−1^. Nitrogen was used as the nebulizer and collision gas.

### Statistical analysis

Principal Component analysis (PCA) was performed by SIMCA, version 14.1. Orthogonal partial least squares discriminant analysis (OPLS-DA) was used to filter out the orthogonal variables in the metabolites which were not related to the classification variables, and the differences between the metabolites were obtained. Seven cyclic cross-validation and 200 response sequencing tests were used to prevent the model from over-fitting and evaluate the effectiveness of the model.

The *t*-test was used for the analysis of differences to obtain the *P*-value. The variable importance in the projection (VIP) scores were obtained by OPLS-DA model. Variables with VIP ˃1 and *P* ˂0.05 were considered differentially expressed metabolites. SPSS 22.0 was used to analyze the fold change (FC). Log_2_FC > 0 and log_2_FC < 0 represented up-regulation and down-regulation, respectively.

## Results and discussion

### Morphology and structural characterization of MSNs and Ace@MSNs

**Fig. 1:**
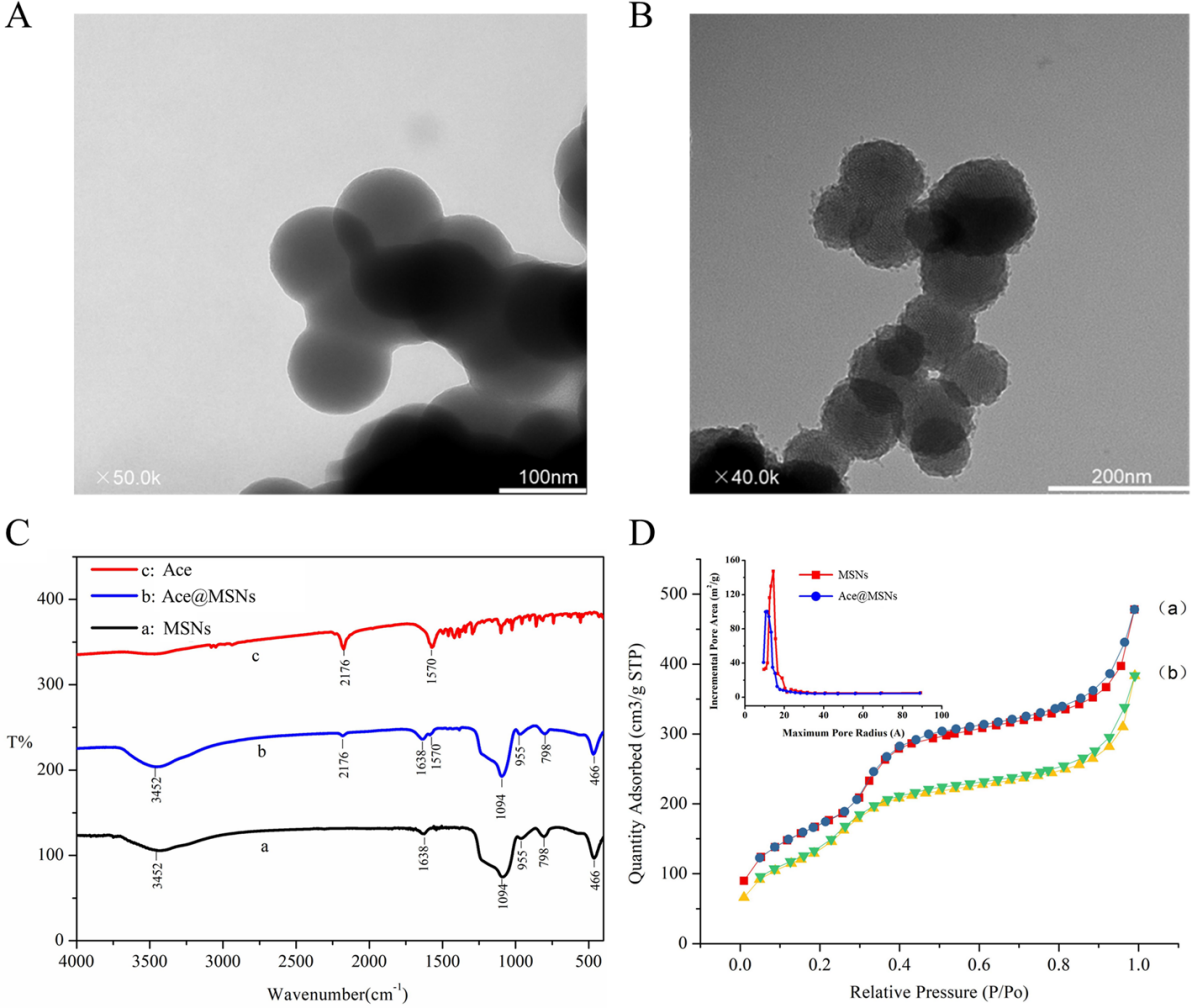
Comparison of MSNs-C and MSNs. TEM images of **A** MSNs-C and **B** MSNs. **C** Infrared spectra of MSNs (a), Ace@MSNs (b) and unloaded ace (c), **D** nitrogen adsorption curves for adsorption curve of MSNs (squares), desorption curve of MSNs (circles), adsorption curve of Ace@MSNs (up triangles) and desorption curve of Ace@MSNs (down triangles) and (inset) pore size distribution of MSNs (squares) and Ace@MSNs (circles)

TEM imaging of the commercial MSNs-C (Fig. 1A) and the MSNs manufactured according to our procedure (Fig. 1B) clearly showed that both kinds of nano silicas were uniformly spherical with particle sizes between 100 and 200 nm. The interior pore structure can be seen in the MSNs.

The FTIR spectra of Ace, MSNs and Ace@MSNs are shown in Fig. 1C. The characteristic absorption peak at 1094 cm^−1^ in MSNs and Ace@MSNs was attributed to the Si–O-Si antisymmetric stretching vibration. This peak is the characteristic peak of amorphous silica, which indicates that the MSNs are amorphous silica. The typical peaks at 2176 and 1570 cm^−1^ in Ace and Ace@MSNs, corresponding to C≡N and C=N stretching, were absent in the MSNs samples, implying that acetamiprid was successfully loaded into the MSNs. There was no shift phenomenon and new absorption peak in the whole peak diagram. This indicates that acetamiprid and MSNs have a physical adsorption or weak interaction. The Brunauer–Emmett–Teller (BET)-specific surface area for MSNs was calculated to be 645 m^2^ g^−1^ and the internal aperture was 1.44 nm, which was decreased to 603 m^2^ g^−1^ and 1.12 nm respectively for acetamiprid (Ace)-loaded samples (Ace@MSNs) (Fig. 1D), likely due to the occupation of nanochannels by acetamiprid molecules.

Changing the time allowed for drug loading of Ace@MSN showed that the adsorption capacity of MSNs for acetamiprid was the highest at 2 h, and gradually decreased with time after 2 h (Fig. S1). Therefore, 2 h was chosen as the adsorption time. When a 10 µg g^−1^ solution of acetamiprid in ultrapure water was mixed with 10 g L^−1^ MSNs for 2 h, the MSNs had an adsorption capacity for acetamiprid of 1.73 mg g^−1^, while in dichloromethane it was 1.78 mg g^−1^ (Fig. S2). However, dichloromethane has a certain toxicity. Therefore, ultrapure water was selected as the solvent of acetamiprid in the follow-up experiments. With an increase in the initial concentration, the MSNs adsorption capacity for acetamiprid increased. The adsorption capacity for acetamiprid of the synthesized MSNs in ultrapure water could reach 354.01 mg g^−1^ when the acetamiprid was added a concentration of 7.2 g g^−1^.

To study the adsorption rate and mechanism, pseudo-first order and pseudo-second order kinetic models were used to fit the experimental data of acetamiprid loading by MSNs. The pseudo-first order dynamic equation was calculated according to the following equation:

$$\log \left( {Q_{{\text{e}}} - Q_{{\text{t}}} } \right) = \log Q_{e} - \frac{{{\text{K}}_{1} }}{2.303}{\text{t}}$$

The pseudo-second order dynamic model was calculated according to the following equation:

$$\frac{{\text{t}}}{{Q_{{\text{t}}} }} = \frac{1}{{K_{2} Q_{e}^{2} }} + \frac{1}{{Q_{e} }}{\text{t}}$$

where K_1_ is the adsorption rate constant of the quasi-first order equation; K_2_ is the adsorption rate constant of the quasi-second order equation; Q_e_ (mg g^−1^) is the adsorption capacity of the adsorbent at equilibrium (the maximum adsorption capacity was measured); Q_t_ (mg g^−1^) is the adsorption time at t min, the adsorption capacity of adsorbent; and t (h) is the adsorption time of the adsorbent.

The data showed that the fitting coefficients (*R*^2^) of the pseudo-second order kinetic model were higher than those of the pseudo-first order kinetic model. In addition, the Q_e_ value of the pseudo-second order kinetic model was 1.21 mg g^−1^, which was close to the real experimental value of 1.6074 mg g^−1^. The results showed that the adsorption behavior of MSNs for acetamiprid conforms to the pseudo-second order kinetic model.

The loading behavior of acetamiprid on MSNs was studied according to the Langmuir and Freundlich adsorption isotherm equations. The Langmuir adsorption isotherm was calculated according to the following equation:

$$\frac{{C_{e} }}{{Q_{e} }} = \frac{1}{{Q^{0} b}} + \frac{{C_{e} }}{{Q^{0} }}$$

The Freundlich adsorption isotherm was calculated according to the following equation:

$$\lg Q_{e} = \lg K + \frac{1}{n}\lg C_{e}$$

where C_e_ (mg L^−1^) is the concentration of solution at the equilibrium of adsorption; Q_e_ (mg L^−1^) is the adsorption capacity at equilibrium; b is the Langmuir adsorption isotherm equation adsorption constant; Q^0^ (mg L^−1^) is the saturated adsorption capacity of the adsorbent; K is the Freundlich adsorption isotherm equation adsorption coefficient; and n is the Freundlich adsorption isotherm equation adsorption constant.

The fitting parameter (*R*^2^) of Freundlich model was more than 0.99, which indicated that the Freundlich adsorption kinetic equation could describe the adsorption process of acetamiprid by MSNs. However, the Q^0^ fitted by the Langmuir model was not very different from the experimental value, so there may be multiple adsorption modes coexisting in the MSNs.

### Insecticidal activity of Ace@MSNs

In order to explore the application potential of nano-pesticides in agricultural pest control, tea aphid was used as a model insect and treated with Ace@MSNs, acetamiprid (Ace) and a commercial preparation of acetamiprid (Ace-C). The results showed that the lethal rates of all the pesticide treatments were dose dependent and their insect killing efficiency were as follows: Ace@MSNs > Ace > Ace-C. As shown in Table 1, the LC_50_ of Ace@MSNs was 4.687 mg L^−1^ that was remarkably lower than the LC_50_ of Ace and Ace-C (7.713 and 14.08 mg L^−1^ respectively), signifying the enhanced toxicity of Ace@MSNs to tea aphid compared to the free Ace and the commercialized Ace-C product.

**Table 1:** Insecticidal activity of different pesticides (aphids)

Treatment	Toxicity fitting equation	LC_50_	95% confidence interval	*R*^2^
Ace-C	Y = 1.69 + 1.47x	14.08	10.245—18.564	0.924
Ace@MSNs	Y = 0.61 + 1.02x	4.687	1.805—7.523	0.843
Ace	Y = 0.87 + 1.01x	7.713	3.514—11.731	0.847

### Absorption, metabolism and distribution of Ace@MSNs in tea plant

After 10 days of continuous absorption in the pesticide-containing nutrient solution, the absorption rates of Ace-C and Ace@MSNs were determined, before the plants were transferred to nutrient solution lacking the pesticides. The plants were grown for 20 days to determine how the Ace-C and Ace@MSNs pesticides were distributed and metabolized by the tea plants.

**Fig. 2:**
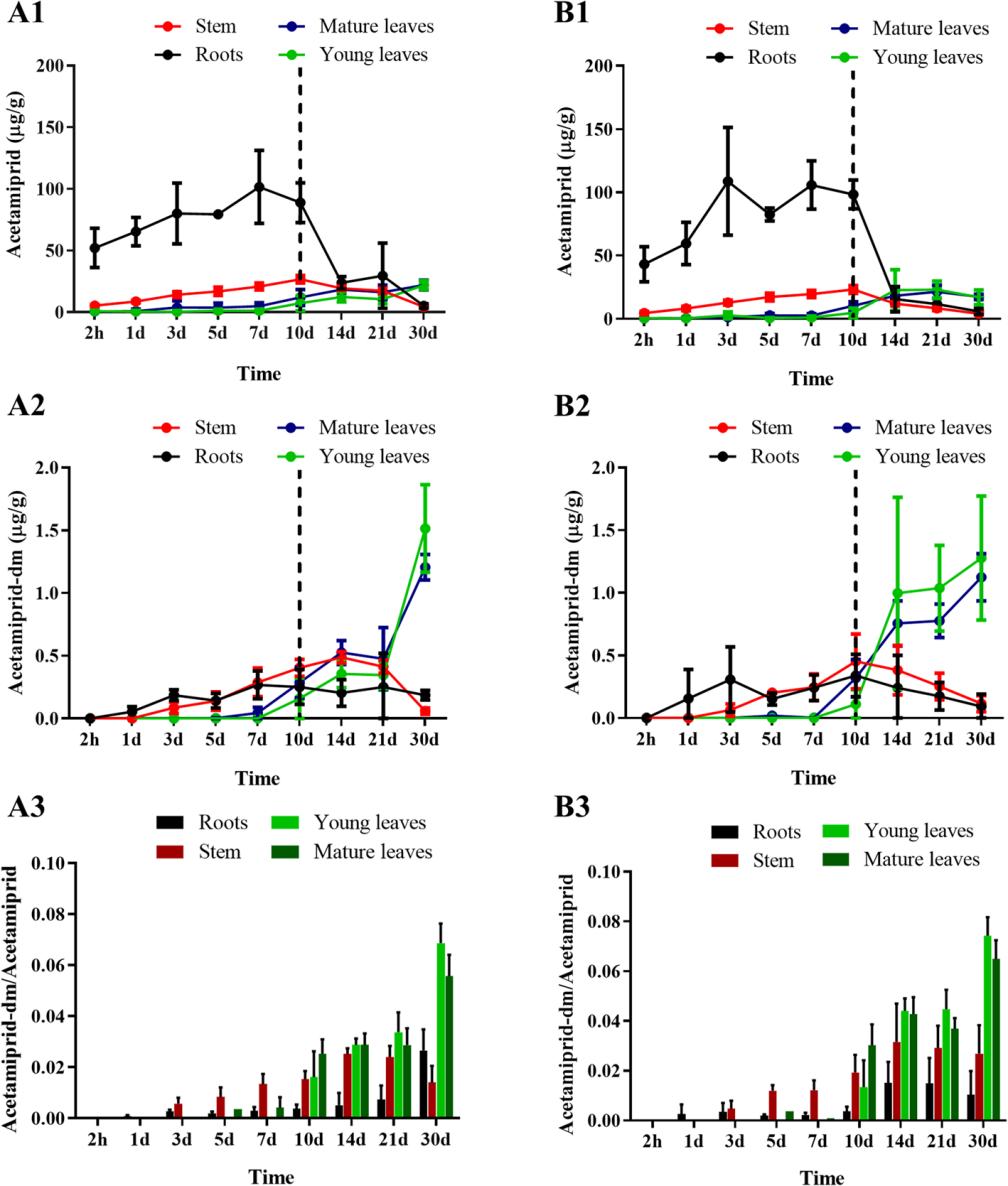
Concentration of (1) acetamiprid and (2) dimethyl-acetamiprid in different parts of tea saplings over time when treated with (**A**) Ace-C or (**B**) Ace@MSNs. Dashed lines represent when the tea saplings were transferred to blank nutrient solution; error bar represents standard deviation (*n* = 6). (3) Ratio of dimethyl-acetamiprid to acetamiprid in various parts of tea tree (A3: Ace-C, B3: Ace@MSNs)

The change in acetamiprid concentration over time in different parts of the tea saplings are shown in Fig. 2A1-B1. In the absorption stage (0–10 days), the concentration of acetamiprid in the roots of tea plants treated with Ace-C and Ace@MSNs showed similar trends, reaching two high concentration points on the 3^rd^ and 7^th^ days.

The acetamiprid content in the roots of tea plants treated with Ace-C reached the maximum on the 7^th^ day, while those treated with Ace@MSNs reached the maximum on the 3^rd^ day. After 10 days of absorption, the accumulated acetamiprid concentration in tea roots was 88.89 µg g^−1^ with Ace-C treatment and 98.30 µg g^−1^ with Ace@MSNs treatment. During the absorption stage, acetamiprid concentration in stems increased with time. In young leaves, acetamiprid concentration was low and did not change significantly over the first 7 days, but increased slightly by the 10^th^ day. Acetamiprid concentration in mature leaves increased with time. Acetamiprid concentration in roots and stems of the two treatment groups decreased with time within 20 days of entering the metabolic stage, and reached low values in roots within 4 days of metabolism. In the metabolic stage, acetamiprid concentrations in the young and mature leaves of tea plants treated with Ace-C showed an overall upward trend, and then decreased by day 21. Acetamiprid concentrations in the young mature old leaves of tea plants treated with Ace@MSNs increased with time, and then decreased on day 30. After 20 days of metabolism, the young and mature leaves of the two treatment groups retained 21.89 and 22.12 µg g^−1^ (Ace-C treatment group), 17.00 and 17.34 µg g^−1^ (Ace@MSNs treatment group). The average retention of acetamiprid in the Ace@MSNs treatment group was slightly lower than that in Ace-C treatment group.

The content of the acetamiprid metabolite dimethyl-acetamiprid was determined (Fig. 2A2-B2). During the experimental treatment of tea sapling, dimethyl-acetamiprid was detected in the root from day 1, and dimethyl-acetamiprid was detected in the stem by the third day. During the whole experimental period, the concentration of dimethyl-acetamiprid in the root showed no obvious change. During the absorption stage (0–10 days), the concentration of dimethyl-acetamiprid in stems increased with time, but decreased with time during the metabolic stage. Dimethyl-acetamiprid was detected in young and mature leaves by the 10^th^ day, and the concentration of dimethyl-acetamiprid increased with time. The concentrations of dimethyl-acetamiprid in young and mature leaves on the 14^th^ and 21^st^ days in the Ace@MSNs treatment group were higher those that in the Ace-C treatment group, and the difference was significant (*P* < 0.05).

The ratio of the of dimethyl-acetamiprid content to acetamiprid content was further compared (Fig. 2A3-B3). Ratios of all treatment groups were lower than 0.08. The ratio of metabolite to parent compound in the root of the tea plant did not change much. The ratio of metabolite to parent compound in the stem increased with time and then stabilized. On day 30, the ratio of metabolite to parent compound in the Ace-C treatment group decreased. On the 14^th^ and 21^st^ days, the ratio of metabolite to parent compound in the Ace@MSNs treatment group was significantly higher than that in the Ace-C treatment group. It is possible that the rate of metabolism of acetamiprid to dimethyl-acetamiprid in the Ace@MSNs treatment group was faster than that in the Ace-C treatment group at the later metabolic stage.

Fresh tea leaves are the raw materials for commercial tea production. Generally, younger leaves make a higher grade commercial tea. In this study, we compared the cumulative concentrations of acetamiprid and its metabolite dimethyl-acetamiprid in young leaves and mature leaves between the two treatment groups (Fig. S3). The results showed that the acetamiprid concentration in mature leaves and young leaves increased continuously during the absorption stage and that the acetamiprid concentration in mature leaves was higher than that in young leaves. During the metabolic stage, the acetamiprid concentration in mature leaves was higher than that in young leaves in the Ace-C treatment group, and reached the highest level on day 21, while the acetamiprid concentration in mature leaves was lower than that in young leaves in the Ace@MSNs treatment group, and reached the highest level on day 30. In the absorption stage, dimethyl-acetamiprid could be detected in some old leaves on the 5^th^ and 7^th^ day, and in tender leaves from the 10^th^ day. In the Ace-C treatment group, the concentration of dimethyl-acetamiprid increased with time, and the concentration of dimethyl-acetamiprid in the mature leaves was lower than that in the young leaves. On day 30, the concentration of dimethyl-acetamiprid in the mature leaves was lower than that in the young leaves, and there was a significant difference. In the Ace@MSNs treatment group, the average concentration of dimethyl-acetamiprid increased significantly after 10 days, and the concentration in young leaves was higher than that in mature leaves. There was no significant difference in the content of young leaves and mature leaves at all time points. Therefore, it can be preliminarily inferred that there is no significant difference in the distribution of metabolites between Ace-C and Ace@MSNs in the young or mature leaves.

**Fig. 3:**
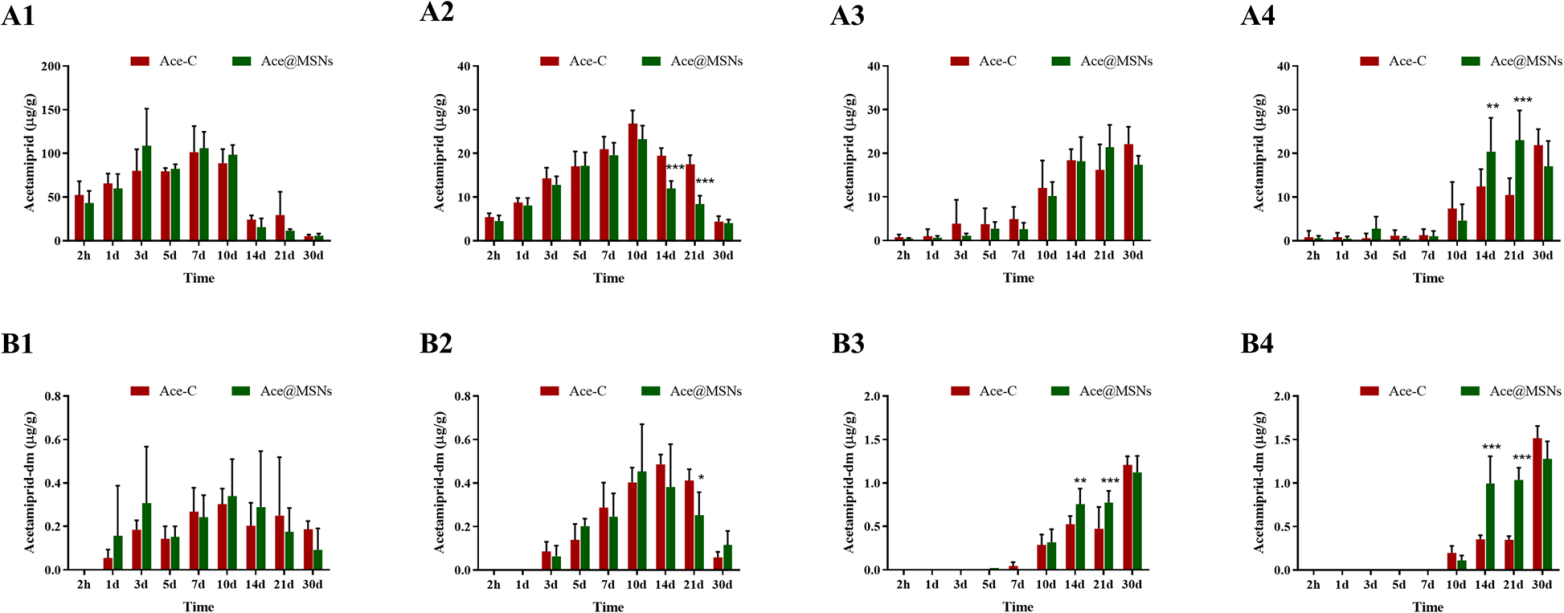
Acetamiprid concentration (**A1**: root, **A2**: stem, **A3**: mature leaves, **A4**: young leaves) and dimethyl-acetamiprid concentration (**B1**: root, **B2**: stem, **B3**: mature leaves, **B4**: young leaves) in different parts of tea saplings

The concentrations of acetamiprid and dimethyl-acetamiprid in different parts of tea saplings were compared under different treatments of Ace@MSNs and Ace-C (Fig. 3). During the absorption stage, acetamiprid concentrations in roots and stems of tea saplings in different treatment groups increased over time but decreased over time during the metabolic stage. On days 14 and 21, acetamiprid concentrations in stems of tea saplings in the Ace-C treatment group were higher than those in the Ace@MSNs treatment group, and there were significant differences. The concentration of acetamiprid in the young leaves during the absorption stage did not change much in the first 7 days, but increased significantly by the 10^th^ day, and also increased with time on the 20^th^ day of the metabolic stage. The concentration of acetamiprid in the young leaves in the Ace-C treatment group was lower than that in the Ace@MSNs group only on the 14^th^ and 21^st^ days, and the differences were significant. During the whole 30 days of the experiment, the acetamiprid concentration in the mature leaves of tea saplings in different treatment groups increased with time.

During the absorption stage, the concentration of dimethyl-acetamiprid in roots reached its highest level on day 10, and then gradually decreased over time in the metabolic stage. The concentration of dimethyl-acetamiprid in the stem also increased and then decreased with time. In the stems, the concentration of dimethyl-acetamiprid was significantly higher in plants in the Ace-C treatment group than those in the Ace@MSNs treatment group on day 21 during metabolic stage. The concentrations of dimethyl-acetamiprid in the mature and young leaves of plants in the Ace@MSNs treatment group were significantly higher than those in the Ace-C treatment group on days 14 and 21, but by day 30, the concentrations of dimethyl-acetamiprid in the mature and young leaves of the Ace@MSNs treatment group were lower than those of the Ace-C treatment group.

The results showed that the acetamiprid concentration in the stem of tea saplings treated with Ace@MSNs was lower than that in the Ace-C treatment group, while the acetamiprid concentration in the young leaves of tea saplings treated with Ace@MSNs was higher than that in the Ace-C treatment group, and these differences were significant on the 14^th^ and 21^st^ days. We speculate that Ace@MSNs are more easily transported to the leaves, especially the young leaves, through the stems during the metabolic stage than the Ace-C. It is important to note that the tender leaves are the most vulnerable parts to insect pests. Therefore, the use of Ace@MSNs can better protect the tea leaves from pests.

### Differentially expressed metabolites of tea leaves under Ace@MSNs and Ace-C treatments

The absorption, metabolism and distribution results for this nano-pesticide in tea plants showed that the absorption and metabolism of Ace@MSNs and Ace-C in the young leaves of tea plants are different on days 14 and 21. In order to study the interaction between the nano-pesticide and tea plants, tea leaves sampled at 2 h and days 7, 14, 21, and 30 were selected for untargeted metabolomics analysis.

The total ion chromatograms (TIC) of tea samples measured by LC–MS in the positive ion mode and the negative ion mode (Fig. S4) were different. In the positive mode, 1528 metabolites were detected, while 1095 metabolites were detected in negative mode. In order to identify metabolic differences between Ace@MSNs treatment and Ace-C treatment, both the positive and negative ion mode datasets were analyzed by multiple pattern recognition.

Principal component analysis (PCA) of the negative ion model data showed that the PCA axes 1 and 2 accounted for 25.3 and 17.6% of the difference at 2 h (Fig. S5a1), 21.1 and 18.1% of the difference at day 7 (Fig. S5b1), 21 and 15.8% of the difference at day 21 (Fig. S5c1), and 20.6 and 14.1% of the difference at day 30 (Fig. S5d1). Combined with PCA (Fig. S5a1-d1) and OPLS-DA (Fig. S5a2-d2), the Ace@MSNs treatment group and the Ace-C treatment group could be distinguished from each other at all timepoints.

For the samples taken at 2 h, the *R*^2^ and Q^2^ values of 200 response ranking tests were 0.914 and -0.232, respectively (Fig. S6a). The *R*^2^ and Q^2^ values for day 7 were 0.984 and -0.0153, respectively (Fig. S6b), for day 21 were 1 and 0.44, respectively (Fig. S6c), and for day 30 were 0.97 and -0.0192, respectively (Fig. S6d). These data proved that the OPLS-DA model was reliable. The OPLS-DA data was used to generate an S-plot (Fig. S7a-d). The points far away from the coordinate axis represent the metabolites with significant differences in content between the Ace@MSNs treatment group and the Ace-C treatment group. The results showed that the metabolites of tea leaves treated with Ace@MSNs were significantly different from those treated with Ace-C on all sampling days.

The statistical analysis and VIP values obtained from OPLS-DA analysis of the data from LC–MS negative ion mode identified 196 metabolites (2 h), 200 metabolites (day 7), 207 metabolites (day 21) and 201 metabolites (day 30) that differed between Ace@MSNs treatment and Ace-C treatment (*P* < 0.05 and VIP > 1), respectively. Among these, the Ace@MSNs treatment group had increased levels of 100 (2 h), 65 (day 7), 100 (day 21), 101 (day 30) metabolites compared with the Ace-C treatment group, and decreased levels of 96 (2 h), 135 (day 7), 107 (day 21), and 100 (day 30) metabolites.

Some of the differentially expressed metabolites were identified as organic acids, amino acids, flavonoid glycosides, saccharides, proanthocyanidins, theaflavins and catechins according to comparison of their retention times to the literature (Tables 2 and 3). After Ace-C treatment, tea saplings metabolites contain glutamic acid, salicylic acid, *p*-coumaric acid, ribonic acid, glutamine, sanguiin H4, PG (34:2), epiafzelechin, epiafzelechin and maltotriose which is not contained in Ace@MSNs treatment group. After Ace@MSNs treatment, tea saplings metabolites contain naringenin diglucoside and maltotriose which is not contained in Ace-C treatment group.

**Table 2:** Identification of some differentially expressed metabolites in negative ion mode

Times	Compounds	C: N	*P*-value	VIP
2 h	glutamic acid	19:0	< 0.0001	3.62641
	salicylic acid	18.3: 0	< 0.0001	3.56076
	*p*-coumaric acid	18:0	< 0.0001	3.53449
	ribonic acid	18:0	< 0.0001	3.53053
	glutamine	17.7: 0	< 0.0001	3.49837
	naringenin diglucoside	0:13.8	< 0.0001	3.0926
7 days	sanguiin H4	14.4: 0	< 0.0001	3.67035
	PG (34:2)	14.1: 0	< 0.0001	3.63607
21 days	epiafzelechin	14.4: 0	< 0.0001	4.7142
30 days	epiafzelechin	14.8: 0	< 0.0001	5.17713
	maltotriose	6.9: 0	0.0404	2.70151
	sanguiin H4	0:8.9	0.0102	3.37827

C: N is the logarithm ratio of the response values of the Ace-C treatment group (C) to the Ace@MSNs treatment group (N)

**Table 3:** Identification of some up and down regulated metabolites in negative ion mode

Times	Compounds	log_2_FC	*P*-value	VIP	Regulation
2 h	catechin	2.5110	0.0008	2.6597	up
	kaempferol 3-*O*-galactosyl-rutinoside	2.5110	0.0009	2.5028	up
	chlorogenic acid	1.6781	0.0235	1.2252	up
	quinic acid	1.6323	0.0083	2.3107	up
	maltose	1.1375	0.0259	1.8251	up
	kaempferol	0.7655	0.0009	1.6271	up
	theacitrin A	0.6781	0.0114	1.3291	up
	EGCG	0.4854	< 0.0001	1.0745	up
	theaflavic acid	0.3785	0.0173	1.0274	up
	maltol	0.3785	0.0235	1.0159	up
	rutin	-2.4060	0.0009	1.9649	down
	shikimic acid	-1.2630	0.0119	2.3071	down
	gluconic acid	-1.2016	0.0190	2.2670	down
	malic acid	-0.6781	0.0105	1.7940	down
	sanguiin H4	-0.6781	0.0654	1.5488	down
	prodelphinidin B2 3 ‘-*O*-gallate	-0.2630	0.0000	1.4792	down
7 days	salicylic acid	2.9069	0.0130	1.7385	up
	myricetin 3-robinobioside	2.7655	0.0111	1.8314	up
	theogallin	2.4060	0.0101	1.6506	up
	ribonic acid	1.2630	0.0192	1.3785	up
	procyanidin C1	0.6781	0.0105	1.5553	up
	naringenin diglucoside	0.5850	0.0131	1.3679	up
	theacitrin A	0.3785	0.0194	1.1190	up
	ascorbic acid	-2.5110	0.0009	3.3694	down
	malic acid	-1.0704	0.0351	2.2448	down
	gallic acid	-1.0000	0.0494	2.3669	down
	isovitexin	-0.9260	0.0473	1.9451	down
	chicoric acid	-0.7655	0.0243	1.2676	down
	theaflavin monogallates	-0.6781	0.0331	1.0135	down
	maltol	-0.5850	0.0165	1.5612	down
	inosine	-0.5850	0.0132	1.5336	down
	eriodictyol 7-*O*-glucoside	-0.5850	0.0143	1.3799	down
	*p*-coumaric acid	-0.4854	0.0203	1.3626	down
	ECG	-0.2630	0.0342	1.0748	down
21 days	maltose	1.3785	0.0328	2.7220	up
	theaflavic acid	0.5850	0.0126	1.9103	up
	eriodictyol 7-*O*-glucoside	0.5850	0.0139	1.7880	up
	ascorbic acid	0.3785	0.0199	1.6530	up
	theaflavate B	0.2630	0.0268	1.2361	up
	maltol	0.2630	0.0347	1.1842	up
	chicoric acid	-1.0704	0.0274	1.5488	down
	1-(sn-glycero-3-phospho)-1D-myo inositol	-0.3785	0.0227	1.4349	down
	naringenin diglucoside	-0.2630	0.0272	1.1772	down
	theasinensin A	-0.2630	0.3022	1.1218	down
	epitheaflagallin 3-*O*-gallate	-0.2630	0.0002	1.0737	down
	epiafzelechin 3-gallate	-0.2630	0.0354	1.0093	down
30 days	theaflavate A	1.9635	0.0994	2.3095	up
	eriodictyol 7-*O*-glucoside	1.0704	0.0256	1.7961	up
	ECG3″Me	0.9260	0.0321	1.4929	up
	digalloylglucose	0.8480	0.0215	1.9096	up
	3-galloylprocyanidinB1	0.6781	0.0271	1.5867	up
	procyanidin C1	0.5850	0.0175	1.8937	up
	eriodictyol 5,3-*O*-di-*O*-glucoside	0.2630	0.0376	1.0166	up
	ascorbic acid	-1.2016	0.0212	2.1219	down
	theacitrin A	-1.0704	0.0524	1.0692	down
	*p*-coumaric acid	-1.0000	0.0446	2.6475	down
	epitheaflagallin 3-*O*-gallate	-0.6781	0.0113	2.1554	down
	chlorogenic acid	-0.5850	0.0360	1.0295	down
	chicoric acid	-0.2630	0.0347	1.0734	down

After 2 h of Ace@MSNs treatment, the contents of catechin, kaempferol 3-*O*-galactosyl-rutinoside, chlorogenic acid, quinic acid and maltose increased significantly compared to the levels in tea saplings treated with Ace-C, while the contents of rutin, shikimic acid and gluconic acid decreased. After 7 days of Ace@MSNs treatment, the content of myricetin 3-robinobioside, salicylic acid and theogallin increased significantly, while the contents of ascorbic acid, malic acid and gallic acid decreased compared to the levels in tea saplings treated with Ace-C. After 21 days of Ace@MSNs treatment, the contents of maltose, theaflavic acid, eriodictyol 7-*O*-glucoside were significantly and differentially increased, while the contents of cichoric acid were differentially decreased. After 30 days of Ace@MSNs treatment, the contents of theaflavate A and eriodictyol 7-*O*-glucoside were significantly increased, while the contents of ascorbic acid, theacitrin A and *p*-coumaric acid were decreased compared to the levels in tea saplings treated with Ace-C.

Principal component analysis (PCA) of positive ion mode data showed that the PCA axes 1 and 2 accounted for 25.7 and 17.5% of the difference at 2 h (Fig. S8a1), 20.5 and 15.7% of the difference at day 7 (Fig. S8b1), and 20.9 and 16.3% of the difference day 30 (Fig. S8c1). Combined with PCA (Fig. S8a1-c1) and OPLS-DA (Fig. S8a2-c2) analysis, the Ace@MSNs treatment group and Ace-C treatment group could be distinguished from each other at 2 h and days 7 and 30.

The *R*^2^ and Q^2^ values of 200-response ranking tests for the 2-h treatment group were 1 and 0.293, respectively (Fig. S9a), for day 7 were 1 and 0.487, respectively (Fig. S9b), and for day 30 were 0.993 and -0.0335, respectively (Fig. S9c). These data proved that the OPLS-DA model was reliable. The OPLS-DA data was used to generate an S-plot (Fig. S10a-c). The points far away from the coordinate axis represent the metabolites with significant differences in content between the Ace@MSNs treatment group and the Ace-C treatment group. The results showed that at 2 h and days 7 and 30, the metabolites of tea leaves treated with Ace@MSNs were significantly different from those treated with Ace-C.

A combination of the statistical analysis and the VIP values obtained from the OPLS-DA analysis of the LC–MS data from positive ion mode identified 294 metabolites (2 h), 356 metabolites (7^th^ day) and 286 metabolites (30^th^ day) that were different between the Ace@MSNs and Ace-C treatment groups (*P* < 0.05 and VIP > 1). The Ace@MSNs treatment group had increased contents of 125 (2 h), 200 (7^th^ day), and 158 (30^th^ day) metabolites and decreased contents of 169 (2 h), 156 (7^th^ day), and 128 (30^th^ day), metabolites compared with the Ace-C treatment groups.

Through preliminary identification using the literature, some of the differentially expressed metabolites were identified as flavonoid glycosides, alkaloids, amino acids, chlorophylls and esters (Table 4). Compared with Ace-C treatment, Ace@MSNs treatment for 2 h significantly increased the contents of theasinensin B and theaflavin digallate, and decreased the contents of malvidin, myricetin 3-rutinoside and theaflavin-3-gallate. Ace@MSNs treatment for 7 days significantly increased the contents of theaflavin-3-gallate, myricetin 3-rutinoside, and myricetin 3-galactoside, and decreased the contents of theasinensin B, methylxanthine and phaeophorbide B. The contents of dihydroxyphenylalanine, myricetin 3-rutinoside and theogallin were significantly increased by Ace@MSNs treatment for 30 days, while the contents of kaempferol 3-arabinoside, theasinensin B, and malvidin were decreased compared with Ace-C treatment.

**Table 4:** Identification of some up and down regulated metabolites in positive ion mode

Times	Compounds	log_2_FC	*P*-value	VIP	Regulation
2 h	theasinensin B	1.5850	0.0005	3.6673	up
	theaflavin digallate	0.2630	0.0006	2.4302	up
	myricetin 3-rutinoside	-2.0000	0.0488	2.0143	down
	malvidin	-1.0704	0.0111	2.6925	down
	theaflavin-3-gallate	-0.5850	0.0006	2.4382	down
	kaempferol 3-arabinoside	-0.3785	0.0203	1.6345	down
	1-deoxy-1-L-theanino-D-fructopyranose	-0.2630	0.0007	1.3310	down
	myricetin 3-galactoside	-0.1375	0.0446	2.0161	down
7 days	theaflavin-3-gallate	1.3785	0.0100	2.3220	up
	myricetin 3-rutinoside	0.7655	0.0254	1.9254	up
	myricetin 3-galactoside	0.7655	0.0239	1.9300	up
	quercetin 3-arabinopyranoside	0.6781	0.0087	2.7492	up
	1-deoxy-1-L-theanino-D-fructopyranose	0.2630	0.0377	2.0276	up
	theasinensin B	-2.0356	0.0122	2.6353	down
	methylxanthine	-1.2016	0.0242	1.9672	down
	phaeophorbide B	-0.6781	0.0199	2.2833	down
	kaempferol 3-arabinoside	-0.4854	0.0258	1.8483	down
	epicatechin-(4β → 8)-epigallocatechin 3-*O*-gallate	-0.3785	0.0161	3.5834	down
	naringenin	-0.2630	0.0842	2.7588	down
	myricetin	-0.2630	0.0495	2.8777	down
30 days	dihydroxyphenylalanine	0.6781	0.0782	2.6903	up
	PC (18:2)	0.3785	0.0203	1.9210	up
	myricetin 3-rutinoside	0.2630	0.0305	1.6220	up
	theogallin	0.2630	0.0266	1.7103	up
	malvidin	-0.7655	0.0191	2.1655	down
	kaempferol 3-arabinoside	-0.6781	0.0372	1.4226	down
	theasinensin B	-0.5850	0.0122	1.3642	down
	epicatechin-(4β → 8)-epigallocatechin 3-*O*-gallate	-0.4854	0.0101	1.3175	down
	PC (16:0)	-0.3785	0.0197	1.8927	down

Metabolites are closely related to metabolic pathways, while secondary metabolic pathways are closely related to tea quality and stress resistance. According to the existing reports, abscisic acid can be used as to induce the expression of genes encoding key enzyme in secondary metabolism, regulating the synthesis of secondary metabolites in response to biotic or abiotic stress [43]. Polyphenols are a major secondary metabolite, and many important proteins are involved in their biosynthesis. For example, anthocyanin plays a key role in oxidative and stress resistance and is produced from phenylpropanoid and flavonoids [44]. Flavanone-3-hydroxylase (F3H) is a key enzyme in the flavonoid pathway, and its overexpression can increase the content of flavonoids in seeds [45]. The accumulation of catechin was positively correlated with the expression of chalcone synthase (CHS) and dihydroflavonol 4-reductase (DER) [46]. This metabolic analysis showed that there are many differences, especially in anthocyanins, flavonoids and catechins, between the Ace@MSNs and Ace-C treatment groups.

## Conclusion

In this study, we have developed a simple and convenient method of synthesizing acetamiprid-loaded porous silica nanoparticles (Ace@MSNs) that may provide environmental safety benefits. The load rate of acetamiprid reached 354.01 mg g^−1^, the insecticidal activity against tea aphids was higher than that of the commercial preparation of Ace-C. The absorption, transport and metabolism of the acetamiprid in the Ace@MSNs and Ace-C preparations across various parts of tea saplings were compared. By 14 and 21 days after Ace@MSNs treatment, a greater amount of acetamiprid was transported upward from the stem to the young leaves and was metabolized into dimethyl-acetamiprid in the leaves. LC-QTOF-MS was used to detect metabolites in tea leaves, and differences in metabolite content were found in tea leaves treated with Ace@MSNs and Ace-C. According to the preliminary identification of the metabolites, some of the differentially expressed compounds were organic acids, amino acids, alkaloids, flavonoid glycosides, saccharides, procyanidins, theaflavins, catechins and esters. The major up-regulated compounds at different time points were naringenin diglucoside, catechin, kaempferol 3-*O*-galactosyl-rutinoside, salicylic acid, myricetin 3-robinobioside, sanguiin H4, theasinensin B, and theaflavin-3-gallate; the major down-regulated compounds were glutamic acid, salicylic acid, *p*-coumaric acid, ribonic acid, glutamine, sanguiin H4, PG (34:2), epiafzelechin, epiafzelechin, myricetin 3-rutinoside, theasinensin B, and methylxanthine. Some of these compounds showed opposite dynamic changes over time. Research on the absorption, transportation and metabolism of nano pesticides in tea plant can provide research ideas for the application of other agricultural products. Only by confirming the safety of nano pesticides in biological application, can nano pesticides be truly applied in agricultural production and reduce the environmental pollution caused by traditional agricultural drugs [47].

## Supplementary Information

**Additional file 1:** **Figure S1.** Effect of adsorption time on adsorption amount. **Figure S2.** Influence of adsorption solvent on adsorption capacity. **Figure S3.** Concentration of (1) acetamiprid and (2) dimethyl-acetamiprid in mature and young leaves of tea saplings treated with (A1, A2) Ace-C and (B1, B2) Ace@MSNs. **Figure S4.** UPLC-Q-TOF-MS TIC diagrams of the QC samples in positive ion mode (A) and negative ion mode (B). **Figure S5.** Multivariate statistical analysis of metabolites detected by LC-MS in positive ion mode (D: Ace-C; N: Ace@MSNs; up: PCA; down: OPLS-DA analysis; a-d: 2 h, 7 d, 21 d, 30 d). **Figure S6.** LC-MS negative ion mode data subjected to the OPLS-DA model and 200 response ranking tests (a: 2 h; b: 7 days; c: 21 days; d: 30 days). **Figure S7.** LC-MS negative ion mode data for tea saplings treated with Ace@MSNs and Ace-C subjected to S-PLOT analysis (a: 2 h; b: 7 d; c: 21 d; d: 30 d). **Figure S8.** Multivariate statistical analysis of metabolites detected by LC-MS in positive ion mode (D: Ace-C; N: Ace@MSNs; up: PCA; down: OPLS-DA; a-c: 2 h, 7 d, 30 d). **Figure S9.** LC-MS positive ion mode data sunjected to OPLS-DA model with a 200 response ranking test (a: 2 h; b: 7 d; c: 30 d). **Figure S10.** LC-MS positive ion mode data from tea spalings treated with Ace@MSNs and Ace-C subjected to S-PLOT analysis (a: 2 h; b: 7 d; c: 30 d).

## Data Availability

The datasets used and analysed during the current study are available from the corresponding author on reasonable request.

## Abbreviations

MSNs: Mesoporous silica nanoparticles
PVPP: Polyvinylpolypyrrolidone
CTAB: Cetyltrimethyl ammonium bromide
GCB: Graphitized carbon black
PSA: Primary secondary amine
TEOS: Tetraethyl orthosilicate
MSNs-C: Commercially available silica nanoparticles
Ace-C: Acetamiprid commercial preparation
Ace@MSNs: Acetamiprid-loaded porous silica nanoparticles
DL: Drug loading
PCA: Principal Component analysis
OPLS-DA: Orthogonal partial least squares discriminant analysis
VIP: The variable importance in the projection
FC: Fold change
