# Supplementary material for: Delivery of acetamiprid to tea leaves enabled by porous silica nanoparticles: efficiency, distribution and metabolism of acetamiprid in tea plants
Source: BMC Plant Biol. 2021 Jul 16;21:337. doi: 10.1186/s12870-021-03120-4 (PMC8283891; doi:10.1186/s12870-021-03120-4)
Supplement: Supplementary file 1 — Additional file 1: Figure S1. Effect of adsorption time on adsorption amount. Figure S2. Influence of adsorption solvent on adsorption capacity. Figure S3. Concentration of (1) acetamiprid and (2) dimethyl-acetamiprid in mature and young leaves of tea saplings treated with (A1, A2) Ace-C and (B1, B2) Ace@MSNs. Figure S4. UPLC-Q-TOF-MS TIC diagrams of the QC samples in positive ion mode (A) and negative ion mode (B). Figure S5. Multivariate statistical analysis of metabolites detected by LC-MS in positive ion mode (D: Ace-C; N: Ace@MSNs; up: PCA; down: OPLS-DA analysis; a-d: 2 h, 7 d, 21 d, 30 d). Figure S6. LC-MS negative ion mode data subjected to the OPLS-DA model and 200 response ranking tests (a: 2 h; b: 7 days; c: 21 days; d: 30 days). Figure S7. LC-MS negative ion mode data for tea saplings treated with Ace@MSNs and Ace-C subjected to S-PLOT analysis (a: 2 h; b: 7 d; c: 21 d; d: 30 d). Figure S8. Multivariate statistical analysis of metabolites detected by LC-MS in positive ion mode (D: Ace-C; N: Ace@MSNs; up: PCA; down: OPLS-DA; a-c: 2 h, 7 d, 30 d). Figure S9. LC-MS positive ion mode data sunjected to OPLS-DA model with a 200 response ranking test (a: 2 h; b: 7 d; c: 30 d). Figure S10. LC-MS positive ion mode data from tea spalings treated with Ace@MSNs and Ace-C subjected to S-PLOT analysis (a: 2 h; b: 7 d; c: 30 d). [file 12870_2021_3120_MOESM1_ESM.docx]

**Supporting informations for *BMC Plant Biology***

**Delivery of acetamiprid to tea leaves enabled by porous silica nanoparticles: Efficiency, distribution and metabolism of acetamiprid in tea plants**

Xinyi Wang^#a^, MinYan^#a^, Jie Zhou^b^, Wei Song^c^, Yu Xiao^c^, Chuanjian Cui^a^, Wanjun Gao^a^, Fei Ke^a^, Jing Zhu^a^, Zi Gu^d^, Ruyan Hou^a*^

^a^ State Key Laboratory of Tea Plant Biology and Utilization; Key laboratory of Food Nutrition and Safety, School of Tea and Food Science & Technology, Anhui Agricultural University, Hefei, 230036, China;

^b^ School of Environmental Science and Engineering, Shanghai Jiao Tong University, 800 Dongchuan Road, Shanghai 200240, China

^c^ Hefei Customs District Technical Center, Anhui Key Lab of Analysis and Detection for Food Safety, Hefei, 230022, China;

^d^ School of Chemical Engineering, The University of New South Wales, Sydney, 2052 NSW, Australia;

^*^ Corresponding author: Ruyan Hou, hry@ahau.edu.cn, Tel: +86-0551-65786765,

ORCID ID: https://orcid.org/0000-0003-4423-694X;

^#^ Xinyi Wang and MinYan contributed equally to this work.

**Content index**

**Fig. S1.** Effect of adsorption time on adsorption amount.

**Fig. S2.** Influence of adsorption solvent on adsorption capacity.

**Fig. S3.** Concentration of (1) acetamiprid and (2) dimethyl-acetamiprid in mature and young leaves of tea saplings treated with (A1, A2) Ace-C and (B1, B2) Ace@MSNs.

**Fig. S4.** UPLC-Q-TOF-MS TIC diagrams of the QC samples in positive ion mode (A) and negative ion mode (B).

**Fig. S5.** Multivariate statistical analysis of metabolites detected by LC-MS in positive ion mode (D: Ace-C; N: Ace@MSNs; up: PCA; down: OPLS-DA analysis; a-d: 2 h, 7 d, 21 d, 30 d).

**Fig. S6.** LC-MS negative ion mode data subjected to the OPLS-DA model and 200 response ranking tests (a: 2 h; b: 7 days; c: 21 days; d: 30 days).

**Fig. S7.** LC-MS negative ion mode data for tea saplings treated with Ace@MSNs and Ace-C subjected to S-PLOT analysis (a: 2 h; b: 7 d; c: 21 d; d: 30 d).

**Fig. S8.** Multivariate statistical analysis of metabolites detected by LC-MS in positive ion mode (D: Ace-C; N: Ace@MSNs; up: PCA; down: OPLS-DA; a-c: 2 h, 7 d, 30 d).

**Fig. S9.** LC-MS positive ion mode data sunjected to OPLS-DA model with a 200 response ranking test (a: 2 h; b: 7 d; c: 30 d).

**Fig. S10.** LC-MS positive ion mode data from tea spalings treated with Ace@MSNs and Ace-C subjected to S-PLOT analysis (a: 2 h; b: 7 d; c: 30 d).

**
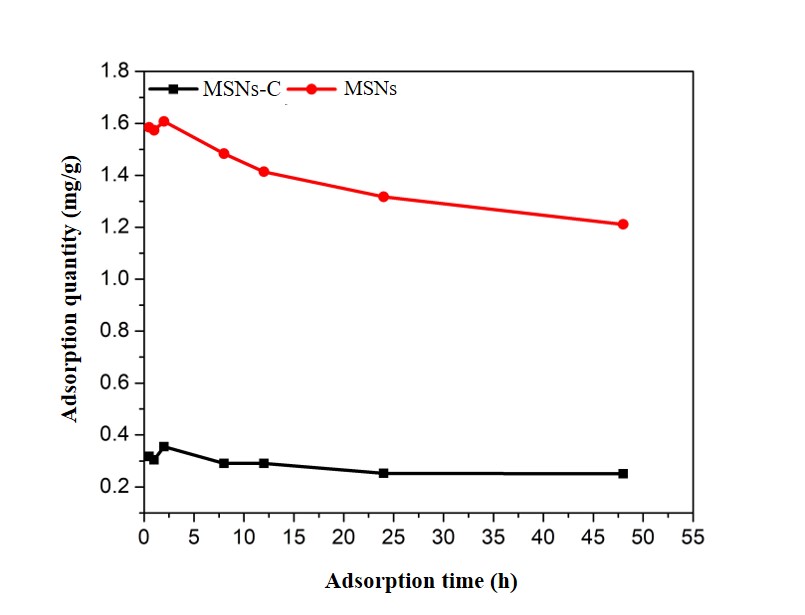
**

**Fig. S1.** Effect of adsorption time on adsorption amount.

**
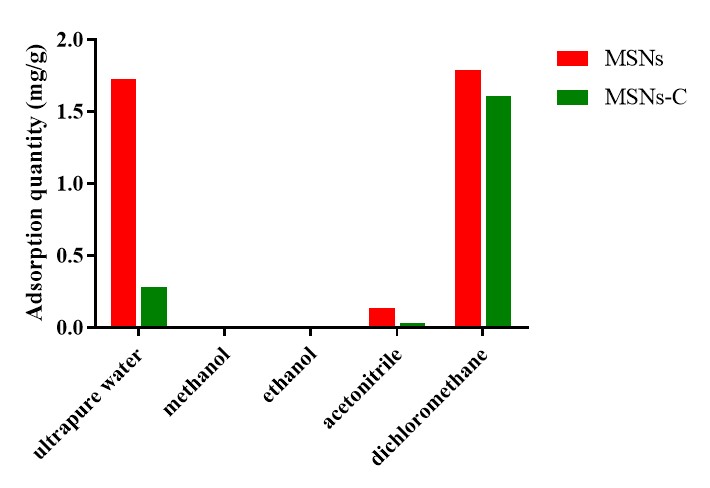
**

**Fig. S2.** Influence of adsorption solvent on adsorption capacity.


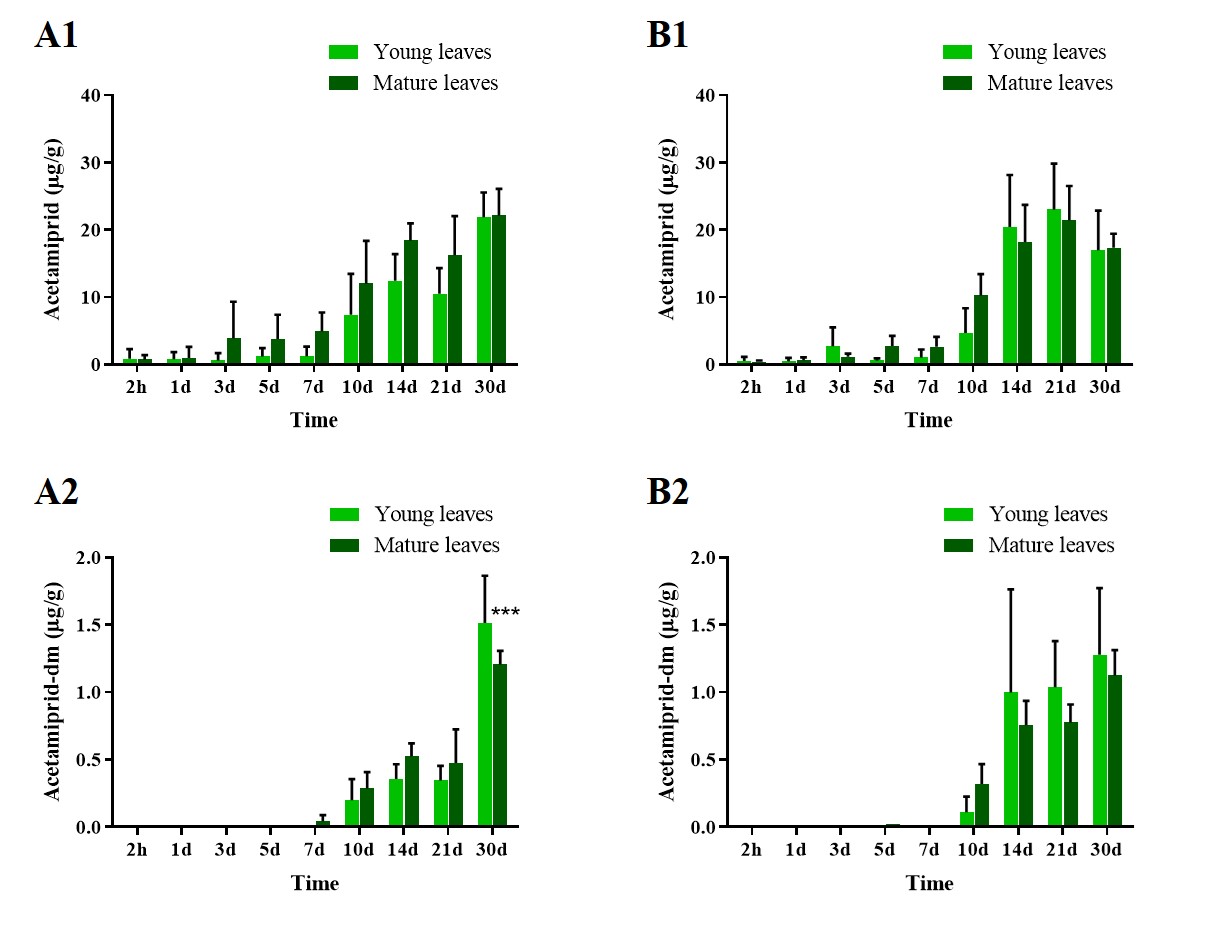


**Fig. S3.** Concentration of (1) acetamiprid and (2) dimethyl-acetamiprid in mature and young leaves of tea saplings treated with (A1, A2) Ace-C and (B1, B2) Ace@MSNs.


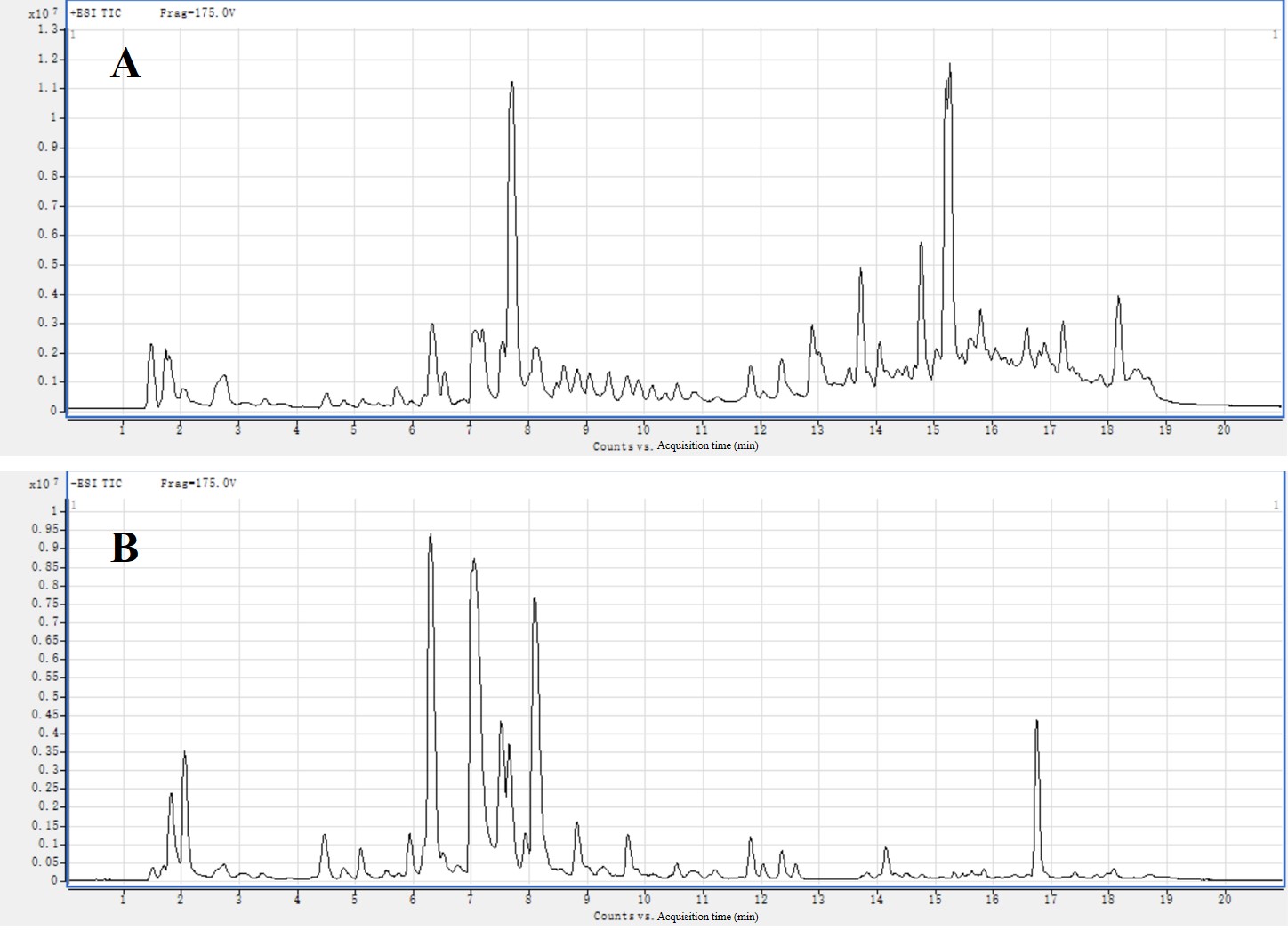


**Fig. S4.** UPLC-Q-TOF-MS TIC diagrams of the QC samples in positive ion mode (A) and negative ion mode (B).


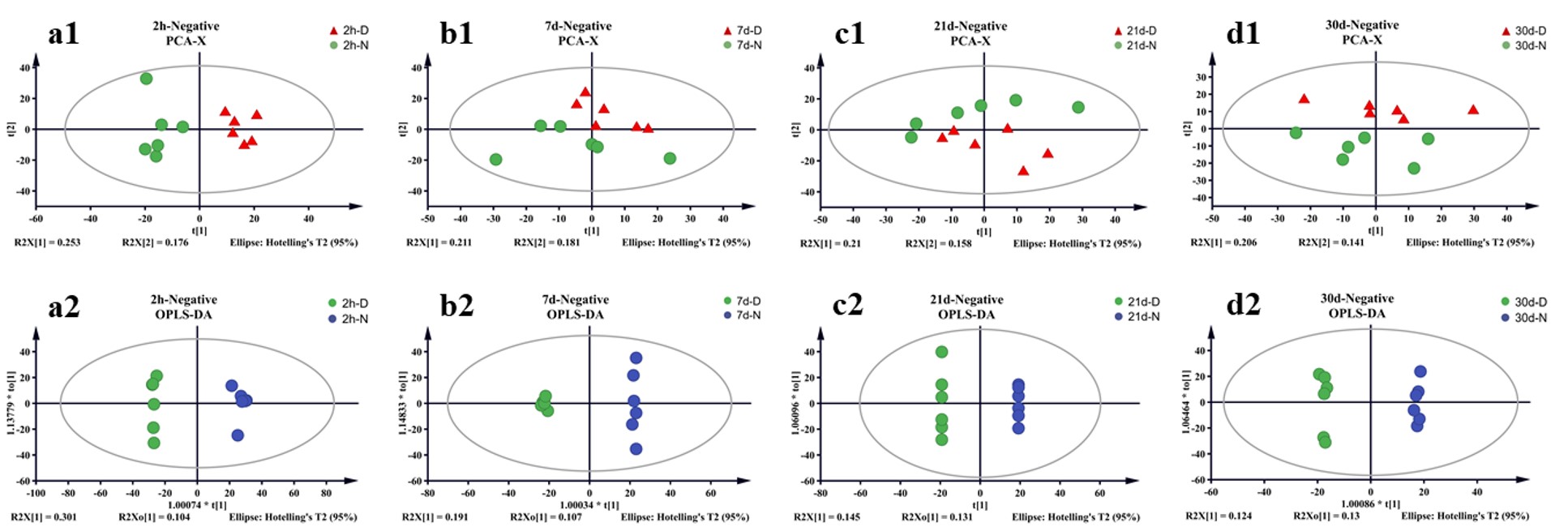


**Fig. S5.** Multivariate statistical analysis of metabolites detected by LC-MS in positive ion mode (D: Ace-C; N: Ace@MSNs; up: PCA; down: OPLS-DA analysis; a-d: 2 h, 7 d, 21 d, 30 d).


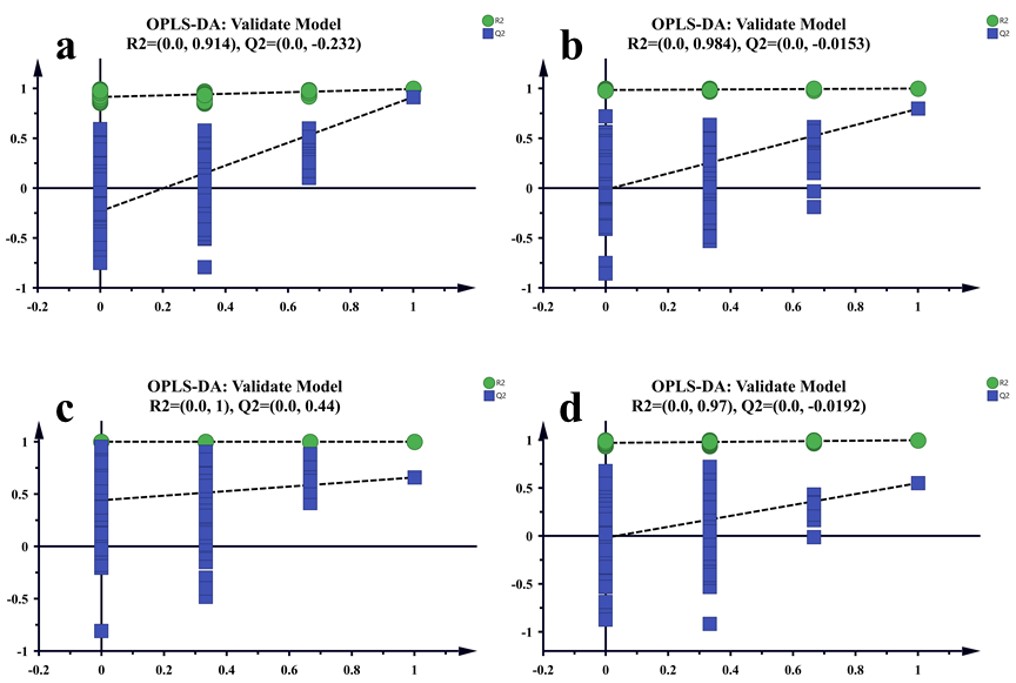


**Fig. S6.** LC-MS negative ion mode data subjected to the OPLS-DA model and 200 response ranking tests (a: 2 h; b: 7 days; c: 21 days; d: 30 days).


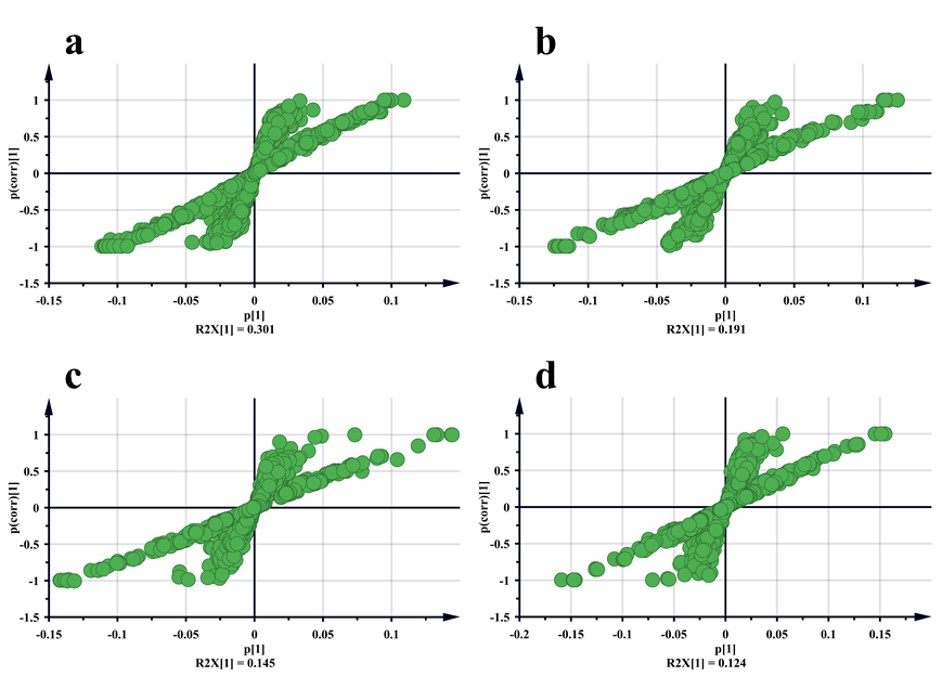


**Fig. S7.** LC-MS negative ion mode data for tea saplings treated with Ace@MSNs and Ace-C subjected to S-PLOT analysis (a: 2 h; b: 7 d; c: 21 d; d: 30 d).


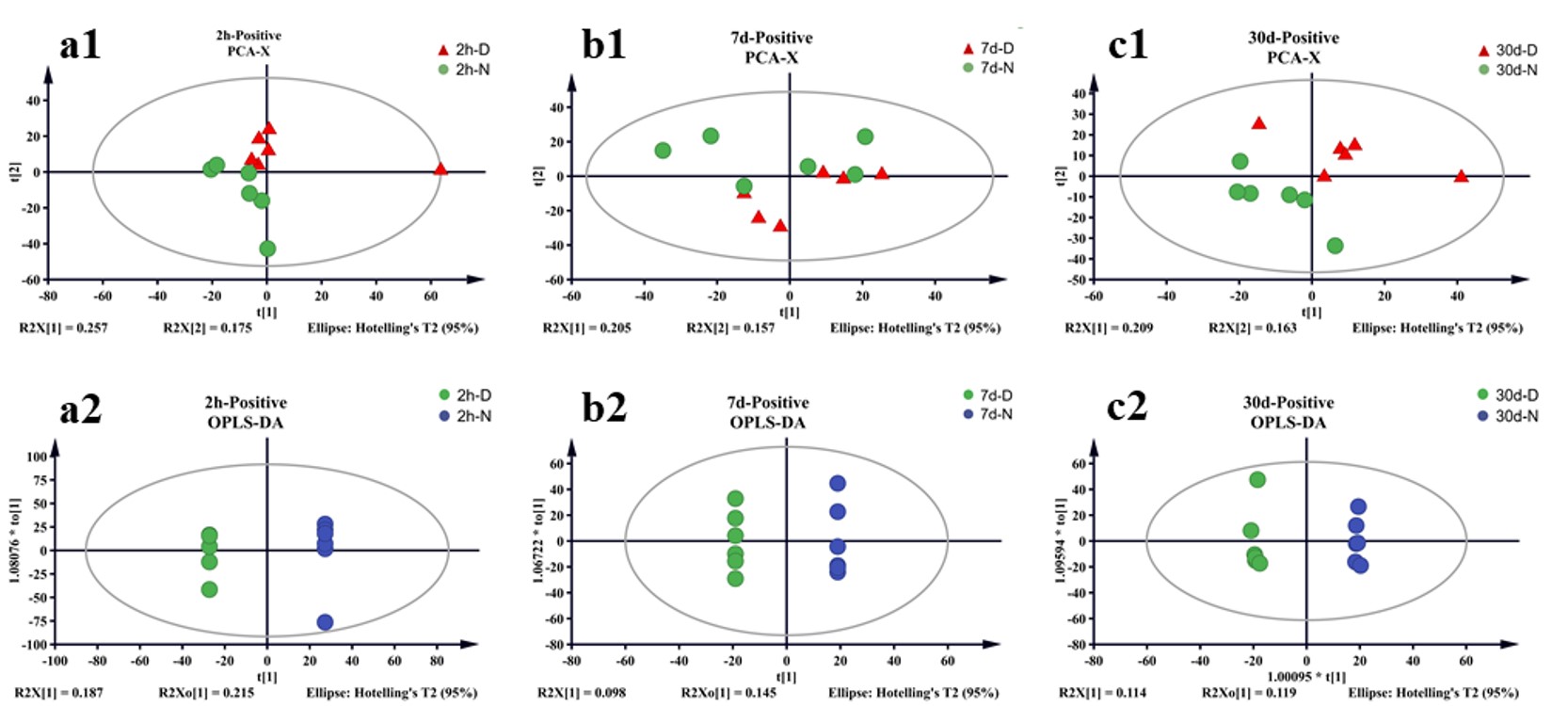


**Fig. S8.** Multivariate statistical analysis of metabolites detected by LC-MS in positive ion mode (D: Ace-C; N: Ace@MSNs; up: PCA; down: OPLS-DA; a-c: 2 h, 7 d, 30 d).


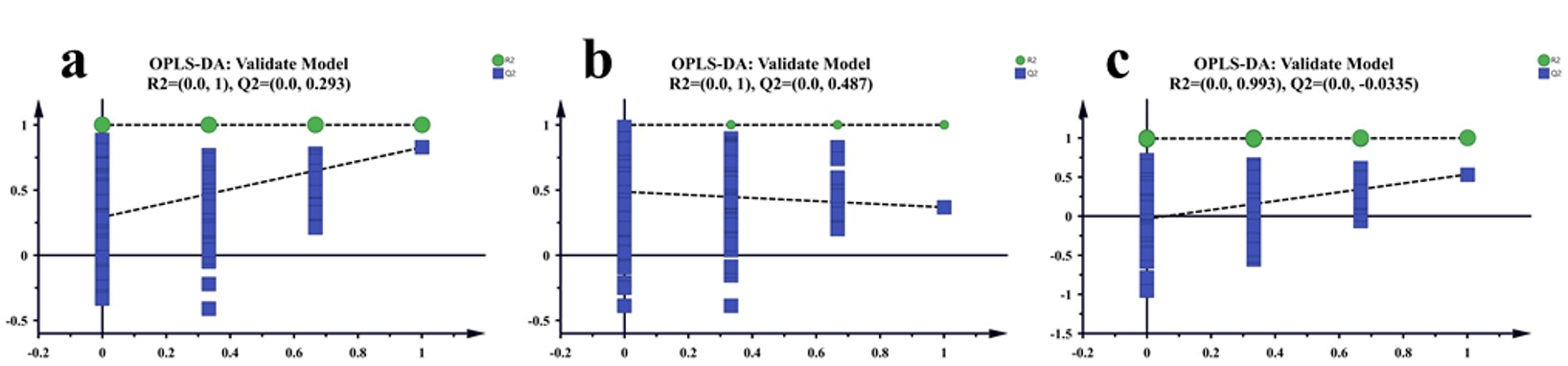


**Fig. S9.** LC-MS positive ion mode data sunjected to OPLS-DA model with a 200 response ranking test (a: 2 h; b: 7 d; c: 30 d).


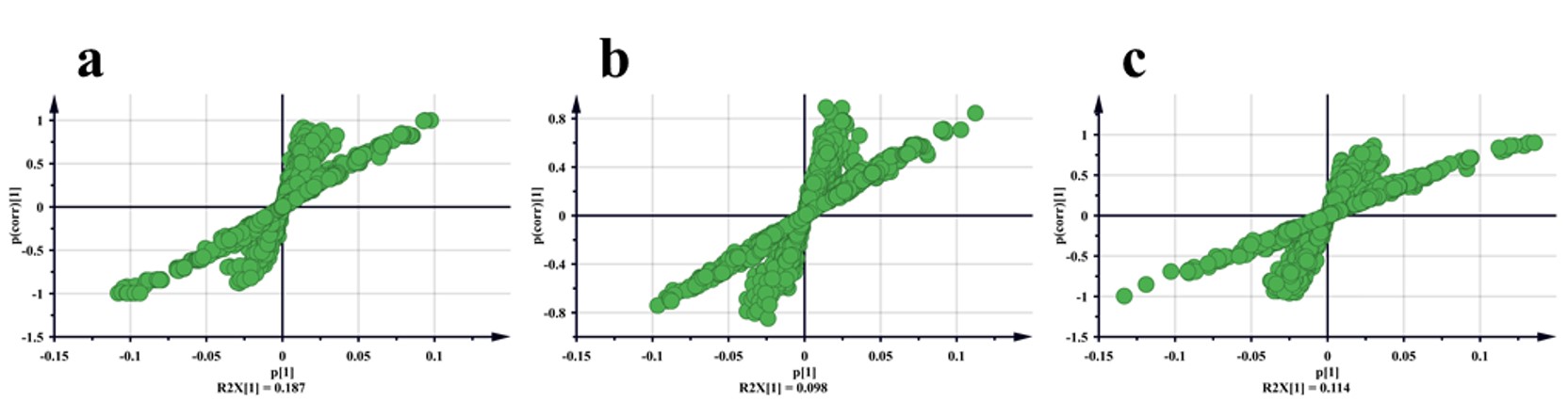


**Fig. S10.** LC-MS positive ion mode data from tea spalings treated with Ace@MSNs and Ace-C subjected to S-PLOT analysis (a: 2 h; b: 7 d; c: 30 d).
